# Lifestyle and sociodemographic risk factors for stillbirth by region of residence in South Australia: a retrospective cohort study

**DOI:** 10.1186/s12884-024-06553-5

**Published:** 2024-05-15

**Authors:** Anneka Bowman, Thomas Sullivan, Maria Makrides, Vicki Flenady, Emily Shepherd, Karen Hawke, Deanna Stuart-Butler, Cathy Leane, Philippa Middleton

**Affiliations:** 1 Aboriginal Communities and Families Health Research Alliance (ACRA), Adelaide, Australia; 2 South Australian Health and Medical Research Institute, North terrace, Adelaide, Australia; 3 University of Adelaide, Adelaide, Australia; 4 Flinders University, Adelaide, Australia; 5 Centre for Research Excellence in Stillbirth, Mater Research Institute, The University of Queensland, Brisbane, Australia; 6 Aboriginal Health Division of the Women’s and Children’s Health Network, Adelaide, Australia

**Keywords:** Stillbirth, Perinatal death, Environment, Pregnancy, Reproductive health, Risk

## Abstract

**Background:**

Stillbirth rates remain a global priority and in Australia, progress has been slow. Risk factors of stillbirth are unique in Australia due to large areas of remoteness, and limited resource availability affecting the ability to identify areas of need and prevalence of factors associated with stillbirth. This retrospective cohort study describes lifestyle and sociodemographic factors associated with stillbirth in South Australia (SA), between 1998 and 2016.

**Methods:**

All restigered births in SA between 1998 ad 2016 are included. The primary outcome was stillbirth (birth with no signs of life ≥ 20 weeks gestation or ≥ 400 g if gestational age was not reported). Associations between stillbirth and lifestyle and sociodemographic factors were evaluated using multivariable logistic regression and described using adjusted odds ratios (aORs).

**Results:**

A total of 363,959 births (including 1767 stillbirths) were included. Inadequate antenatal care access (assessed against the Australian Pregnancy Care Guidelines) was associated with the highest odds of stillbirth (aOR 3.93, 95% confidence interval (CI) 3.41–4.52). Other factors with important associations with stillbirth were plant/machine operation (aOR, 1.99; 95% CI, 1.16–2.45), birthing person age ≥ 40 years (aOR, 1.92; 95% CI, 1.50–2.45), partner reported as a pensioner (aOR, 1.83; 95% CI, 1.12–2.99), Asian country of birth (aOR, 1.58; 95% CI, 1.19–2.10) and Aboriginal/Torres Strait Islander status (aOR, 1.50; 95% CI, 1.20–1.88). The odds of stillbirth were increased in regional/remote areas in association with inadequate antenatal care (aOR, 4.64; 95% CI, 2.98–7.23), birthing age 35–40 years (aOR, 1.92; 95% CI, 1.02–3.64), Aboriginal and/or Torres Strait Islander status (aOR, 1.90; 95% CI, 1.12–3.21), paternal occupations: tradesperson (aOR, 1.69; 95% CI, 1.17–6.16) and unemployment (aOR, 4.06; 95% CI, 1.41–11.73).

**Conclusion:**

Factors identified as independently associated with stillbirth odds include factors that could be addressed through timely access to adequate antenatal care and are likely relevant throughout Australia. The identified factors should be the target of stillbirth prevention strategies/efforts. SThe stillbirth rate in Australia is a national concern. Reducing preventable stillbirths remains a global priority.

**Supplementary Information:**

The online version contains supplementary material available at 10.1186/s12884-024-06553-5.

## Background

Globally, more than 2.64 million babies are stillborn annually, with the highest rates occurring in low- and middle-income countries (LMICs) [1, 2]. In high-income countries (HICs), preventable stillbirths continue to be of concern, with slow progress towards global targets for rate reduction. To address this issue, the Australian government appointed a Senate Select Committee on Stillbirth Research and Education in 2018 [3]. Their report revealed that Australia had ‘slipped’ in its progress to reduce stillbirth rates in line with targets compared with other HICs. It also demonstrated that babies born to mothers living remotely were more likely to be stillborn than babies born in major cities [4]. In 2020, *Women and Birth* published a series focused on stillbirth in Australia and identified the national action required to decrease rates [5–9]. Rumbold et al. [9] highlighted the impact of inequity on stillbirth rates within select Australian populations, noting particular concern within communities experiencing isolation and socioeconomic disadvantage [9]. Numerous risk factors in disadvantaged communities contribute to the widening gap in health inequality, further hindering stillbirth prevention [10]. This research aims to identify lifestyle and sociodemographic risk factors for stillbirth in South Australia (SA) geographically and to explore these risks according to remoteness.

## Methods

### Study design and setting

This was a retrospective state-wide observational cohort study using the SA perinatal outcomes dataset, including all births from 1998 to 2016 (cohort one). The dataset contains pregnancy outcomes categorised as live birth or stillbirth. The data were obtained anonymously, with all identifying fields removed prior to their provision for research purposes. The study concept, acceptability, methods and interim analysis were presented, reviewed, and approved by the NHMRC Centre for Research Excellence in Stillbirth Indigenous Advisory Committee at two separate timepoints. The final manuscript was reviewed and approved by local SA Indigenous researchers and senior health care advisors prior to submission.

## Materials

In SA, all births are reported by midwives, birth attendants and obstetricians in standardised supplementary birth records. The SA Perinatal Outcomes Unit integrates continuous validation of the dataset by comparing data collected from the supplementary birth records to electronic hospital records at the time of coding. Sociodemographic characteristics and pregnancy and birth outcome data were recorded. Due to the later introduction of BMI to the data collection, analyses involving BMI were restricted to the years 2007–2016 (cohort two). Terminations of pregnancy were excluded.

### Definitions and outcomes

Variable definitions and time periods are provided in Table 1. Information for all births (live or stillborn) ≥ 20 weeks gestational age (GA) of ≥ 400 g at birth are reported. The primary outcome, stillbirth, was defined in line with the standard Australian Institute of Health and Wellbeing definition as the birth of a baby showing no signs of life at ≥ 20 weeks’ completed GA, or ≥ 400 g birthweight where no GA is provided.

**Table 1:** Study variables included, timepoint of collection and definition of each variable

Variable (availability)	Time point of collection	Definition/categorisation
**Study variables**		
Birthing person’s ethnicity (1998–2016)	First antenatal visit (booking visit)	Self-reported Caucasian, Aboriginal, Torres Strait Islander, Aboriginal and Torres Strait Islander or Asian status. Aboriginal and/or Torres Strait Islander status includes identification by Aboriginal or Torres Strait Islander descent, self-identification of community acceptance of Aboriginal and/or TSI status. Births to women recorded as Aboriginal, Torres Strait Islander, and/or Aboriginal were categorised as Aboriginal and/or Torres Strait Islander women for analysis. Women recorded as Asian were categorised as Asian, and women recorded as Caucasian were categorised as Caucasian
Country of birth (1998–2016)	First antenatal visit (booking visit)	Australia, Oceania, Europe/USSR, Middle East/Nth Africa, SE Asia, NE Asia, Southern Asia, Nth America, South/Central America, Africa as reported by women
Statistical areas Level 3 (SA3) areas (1998–2016)	At birth	Place of usual residence data. Australia Bureau of Statistics modified Accessibility and Remoteness Index of Australia (ARIA+) score average for each SA3 area compiled from SA2 area ARIA + scores. SA3 area was assigned on maternal usual place of residence at birth. Areas were classified as; major cities (geographic distance imposes minimal restrictions upon accessibility to the widest range of goods, services and opportunities for social interaction), inner regional areas (geographic distance imposes some restrictions upon accessibility to the widest range of goods, services and opportunities for social interaction), outer regional areas (geographic distance imposes a moderate restriction upon accessibility to the widest range of goods, services and opportunities for social interaction), remote/very remote areas (geographic distance imposes the highest restriction upon accessibility to the widest range of goods, services and opportunities for social interaction).
Adequacy of antenatal care access (1998–2016)	At birth	Adequacy of antenatal care was assessed per pregnancy according to the Australian Clinical Practice Guidelines: Pregnancy Care that recommends nulliparous women have a minimum of 10 antenatal visits, and multiparous women; a minimum of 7 antenatal visits (40). Adequacy was assigned separately by parity (nulliparous and multiparous) stratified by gestational age
Birthing person’s age (1998–2016)	At birth	Categories: 12–19 years, 20–24 years, 25–29 years, 30–34 years, 35–40 years, ≥ 40 years
Marital status (1998–2016)	At birth	Categories: Married/Unmarried (encompasses; never married, widowed, divorced, separated)
Smoking status (1998–2016)	First antenatal visit (booking visit) and again at 20 weeks GA	Non-smokers as self-reported smoking status at booking visit and 20 weeks GA. Women were classified as smokers if any smoking was reported at either visit
Parity (1998–2016)	First antenatal visit (booking visit)	Nulliparous, multiparous
Chronic health medical conditions	At birth	Previous diabetes or chronic hypertension
Parental occupation	Non-birthing person’s occupation at birth, birthing person occupation prior to and/or during pregnancy before ‘home duties’.	One of 13 occupation groups according to the ABS Australia Standard Classification of Occupations (ASCO) first edition
Inter-pregnancy interval		Calculated as the number of months between the previously recorded birth, and date of conception of the current pregnancy (> 6 months, < 6 months).
Birthing person’s BMI (2007–2016)	First antenatal visit (booking visit) measurements	Calculated as weight in kgs divided by height (in meters) squared. Underweight (< 19), healthy (19–24), overweight (25–29) and class 1 obesity (30–34 years), class 2 obesity (35–39 years), morbid obesity (40+)
Anaemia	At any stage during pregnancy	Anaemia diagnosed as maternal Hb < 10gms/100 ml
**Study confounders**		
Obstetric conditions	At birth	Placental abruption, multiple pregnancy, post-term birth (> 41 completed weeks GA)
Prolonged labour	At birth	Labour duration of > 18 h
Past obstetric history	At birth	Previous caesarean section, previous stillbirth
Medical conditions	At birth	Asthma during pregnancy, urinary tract infection during pregnancy
Babies born small for gestational age	After birth	SGA; below the 10th percentile were determined using Australian national birthweight percentiles estimated from a large Australian cohort of infants born between 1997 and 2007 (41)

Rural and remote living at birth status was based on statistical areas level 3 (SA3) data associated with each birth. Australia Bureau of Statistics modified Accessibility and Remoteness Index of Australia (ARIA+) score average for each SA3 area compiled from SA2 area ARIA + scores. The areas were classified as: major cities, inner regional areas, outer regional areas, or remote/very remote areas. When exposure data or variable data were missing, individual births were excluded from the analysis.

### Statistical analysis

Variables were categorised as outlined in Table 1. Categories with < 10 stillbirths per group were reported as ‘< 10’, and crude odds ratios (ORs) concealed. Within multivariable analysis where categories had fewer than five stillbirths, analyses are reported as ‘< 5’. Logistic regression was performed using the statistical software STATA 16 IC [11] to determine associations between potential risk factors and stillbirth, described using odds ratios (ORs) and 95% confidence intervals (95% CIs). Unadjusted and adjusted models were considered, with adjustments made for variables that demonstrated significance during univariate analysis (*p* < 0.001). For each risk factor, adjustment variables included year of birth, adequate antenatal care (ANC) access (adjusted for GA at birth), marital status, ethnicity, smoking status, parity, remote/rural status, age, previous stillbirth, medical conditions (preexisting diabetes, hypertension, anaemia), plurality, interpregnancy interval, insurance status, and obstetric complications (gestational diabetes, gestational hypertension, antepartum haemorrhage [APH]). The cohort was stratified by residential remoteness, and the analysis was repeated using the same adjustment variables (excluding rural/remote status). Factors demonstrating the strongest association with stillbirth odds were further explored to calculate SA-specific population attributable fractions [12] and annual attributable stillbirths per factor (n). The analysis was repeated for cohort two, which was additionally adjusted for BMI (Tables 4 and 5).

## Results

Data were available for 363,933 births in SA including 1,767 stillborn babies following exclusions (Table 2). Birthing people were predominantly Australian born (81%) with 86% of Australian born people identifying as Caucasian. The majority (71%) lived in major cities, followed by inner regional areas (14%), outer regional areas (8%) and remote or very remote areas (6%). During pregnancy, 13.5% of birthing people access less than the recommended number of ANC visits (Australian Clinical Practice Guidelines: Pregnancy Care recommends that nulliparous women have a minimum of 10 and multiparous women have a minimum of 7). Most birthing people were nonsmokers (78%) and gave birth in the Australian public health care system (70%) (Table 2). Cohort two included 201,315 births (918 stillborn babies) between 2007 and 2016.

**Table 2:** Crude analysis, stillbirth rates and demographic information (cohort one)

Variables		Stillbirths	Total births	Rate/1000 births	Crude OR (95% CI)	*p*-value
**Sociodemographic, lifestyle and environmental factors**						
Smoking	*Non-smoker*	1,197	282,737	4.23	Referent	
	*Smoker*	472	76,130	6.20	**1.47 (1.32, 1.63)**	< 0.001
	*Unknown*	98	5,066	19.35	NR	
Insurance type	*Private*	379	109,022	3.48	Referent	
	*Public*	1,388	254,911	5.45	**1.57 (1.40, 1.76)**	< 0.001
Marital status	*Married*	1440	321,088	4.48	Referent	
	*Unmarried*	326	42,737	7.63	**1.70 (1.51, 1.92)**	< 0.001
	*Unknown*	< 10	108	NR†	NR†	
Adequate antenatal care access	*Adequate antenatal care access*	1,090	314,810	3.46	Referent	
	*Inadequate antenatal care access*	677	49,123	13.78	**4.02 (3.65, 4.44)**	< 0.001
Birthing person’s age	*12–19 years*	119	15,838	7.51	**1.71 (1.39, 2.09)**	
	*20–24 years*	298	54,316	5.49	**1.24 (1.07, 1.44)**	
	*25–29 years*	472	106,830	4.42	Referent	
	*30–34 years*	483	117,263	4.12	0.93 (0.82, 1.06)	< 0.001
	*35–39 years*	302	57,622	5.24	**1.19 (1.02, 1.38)**	
	*≥ 40 years*	93	12,064	7.71	**1.75 (1.40, 2.19)**	
Birthing person’s occupation	*Professionals*	169	50,280	3.36	Referent	
	*Managers/Admin*	91	26,607	3.42	1.02 (0.79, 1.32)	
	*Paraprofessionals*	93	22,528	4.13	1.23 (0.95, 1.59)	
	*Tradespersons*	45	11,594	3.88	1.16 (0.82, 1.63)	
	*Clerks*	149	44,340	3.36	1.00 (0.80, 1.25)	
	*Sales and service workers*	228	53,632	4.25	**1.27 (1.03, 1.55)**	
	*Plant and machine operators*	15	1,882	7.97	**2.38 (1.40, 4.05)**	
	*Labourers*	60	12,051	4.98	**1.48 (1.10, 1.99)**	
	*Student*	81	13,106	6.18	**1.84 (1.41, 2.41)**	
	*Pensioner*	10	1,139	8.78	**2.63 (1.38, 4.99)**	
	*Home duties*	504	93,854	5.37	**1.60 (1.34, 1.91)**	
	*Unemployed*	134	16,434	8.15	**2.44 (1.94, 3.06)**	< 0.001
	*Unknown*	188	16,486	11.40	NR	
Non-birthing person’s occupation	*Professionals*	176	50,581	3.48	Referent	
	*Managers/Admin*	187	57,678	3.24	0.93 (0.76, 1.15)	
	*Paraprofessionals*	62	18,511	3.35	0.96 (0.72, 1.29)	
	*Tradespersons*	251	64,480	3.89	1.12 (0.92, 1.36)	
	*Clerks*	44	9,805	4.49	1.29 (0.92, 1.81)	
	*Sales and service workers*	80	20,395	3.92	1.13 (0.86, 1.48)	
	*Plant and machine operators*	92	22,489	4.09	1.18 (0.91, 1.52)	
	*Labourers*	205	47,252	4.34	**1.25 (1.02, 1.53)**	
	*Student*	47	8,081	5.82	**1.68 (1.21, 2.31)**	
	*Pensioner*	21	2,057	10.21	**2.95 (1.87, 4.65)**	
	*Home duties*	< 10	1,476	NR†	NR†	
	*Unemployed*	145	18,454	7.86	**2.27 (1.81, 2.84)**	< 0.001
	*Unknown*	452	42,674	10.59	NR	
Country of birth (birthing person)	*Australia*	1,424	294,863	4.83	Referent	
	*Europe/USSR*	91	20,115	4.52	0.94 (0.76, 1.16)	
	*Middle east/Nth Africa*	37	5,014	7.38	**1.53 (1.09, 2.14)**	
	*SE Asia*	59	14,334	4.12	0.85 (0.65, 1.11)	
	*NE Asia*	26	6,583	3.95	0.82 (0.55, 1.22)	
	*Southern Asia*	69	11,097	6.22	**1.29 (1.01, 1.65)**	
	*Nth America*	< 10	1,725	NR†	NR†	
	*South/Central America*	< 10	1,392	NR†	NR†	
	*Africa*	34	4,318	7.87	**1.64 (1.15, 2.32)**	
	*Oceania*	13	4,450	2.92	0.60 (0.35, 1.04)	
	*Unknown*	< 10	42	NR†	NR†	0.003
Birthing person’s ethnicity	*Caucasian*	1,404	311,232	4.51	Referent	
	*Aboriginal/Torres Strait Islander*	123	10,773	11.42	**2.55 (2.11, 3.08)**	
	*Asian*	151	29,154	5.18	1.15 (0.97, 1.36)	< 0.001
	*Unknown*	89	12,774	6.97	NR	
Remoteness classification	*Major city*	1,210	257,128	4.71	Referent	
	*Inner regional area*	238	51,219	4.65	0.99 (0.86, 1.14)	
	*Outer regional area*	163	30,880	5.28	1.12 (0.95, 1.32)	
	*Remote/Very remote area*	123	22,305	5.51	1.17 (0.97, 1.42)	< 0.001
	*Unknown/interstate*	33	2,401	13.74	NR	
**Obstetric factors**						
Interpregnancy interval*	*> 6 months*	613	150,178	4.08	Referent	
	*< 6 months*	115	23,245	4.95	1.21 (0.99, 1.49)	< 0.001
	*missing*	226	38,591	5.86	NR	
Parity	*Nulliparous*	813	151,919	5.35	**1.30 (1.18, 1.44)**	
	*1–2 previous births*	747	181,823	4.11	Referent	
	*3 + previous births*	207	30,191	6.86	**1.67 (1.43, 1.96)**	< 0.001
Previous stillbirth*	*No previous stillbirth*	905	208,379	4.34	Referent	
	*Previous Stillbirth*	49	3,635	13.48	**3.13 (2.34, 4.19)**	< 0.001
Previous caesarean*	*No previous caesarean*	659	152,792	4.33	Referent	
	*Previous caesarean*	295	60,176	4.93	1.14 (0.99, 1.31)	0.071
Gestational hypertension	*No gestational hypertension*	1,654	336,395	4.92	Referent	
	*Gestational hypertension*	113	27,538	4.10	0.83 (0.69, 1.01)	0.065
UTI during pregnancy	*No UTI during pregnancy*	1,693	354,852	4.77	Referent	
	*UTI during pregnancy*	74	9,081	8.15	**1.71 (1.35, 2.17)**	< 0.001
Multiple pregnancy	*Singleton*	1,610	352,415	4.57	Referent	
	*Multiple*	157	11,518	13.63	**3.01 (2.48, 3.66)**	< 0.001
Prolonged labour (> 18 h)**	*No prolonged labour*	1,444	243,367	5.93	Referent	
	*Prolonged labour*	73	4,310	16.94	**2.89 (2.27, 3.67)**	< 0.001
GDM	*No GDM*	1,698	342,261	4.96	Referent	
	*GDM*	69	21,672	3.18	0.64 (0.50, 0.82)	< 0.001
Placental abruption	*No placental abruption*	1,610	361,640	4.45	Referent	
	*Placental abruption*	157	2,293	68.47	**16.44 (13.84, 19.52)**	< 0.001
Threatened miscarriage/APH (< 20 weeks GA)	*No threatened miscarriage/APH*	1,657	357,602	4.63	Referent	
	*Threatened miscarriage/APH*	110	6,331	17.37	**3.85 (3.35, 4.42)**	< 0.001
SGA	*Not SGA*	1,177	326,547	3.60	Referent	
	*SGA*	590	37,386	15.78	**4.43 (4.01, 4.90)**	< 0.001
GA at birth	*Term*	452	330,508	1.37	Referent	
	*All preterm (< 37 + 0wks)*	1,308	31,321	41.76	**31.82 (28.55, 35.47)**	< 0.001
	*Post-term (≥ 41 + 7wks)*	< 10	2,096	NR†	NR†	
	*Unknown*	< 10	< 10	NR†	NR†	
**Birthing person’s health**						
Asthma	*No asthma*	1,636	339,648	4.82	Referent	
	*Asthma*	131	24,285	5.39	1.12 (0.93, 1.34)	0.221
Pre-existing diabetes	*No pre-existing diabetes*	1,724	361,644	4.77	Referent	
	*Pre-existing diabetes*	43	2289	18.79	**4.00 (2.94, 5.43)**	< 0.001
Chronic hypertension	*No chronic hypertension*	1,721	359,434	4.79	Referent	
	*Chronic hypertension*	46	4499	10.22	**2.15 (1.59, 2.90)**	< 0.001
Anaemia	*No anaemia during pregnancy*	1,597	334,841	4.77	Referent	
	*Anaemia during pregnancy*	170	29,092	5.84	**1.23 (1.04, 1.44)**	< 0.001

†Not publishable due to small numbers.

*Analysis only includes multiparous women **Analysis only includes vaginal births.

The stillbirth rate in SA over the study period was 4.85/1000 births. Stillbirth rates were highest for birthing people who had inadequate ANC access (13.78/1000 births) and those who reported that they (8.78/1000 births) or their non-birthing partner were a ‘pensioner’ (10.21/1000 births). Stillbirths were high among ‘unemployed’ individuals and ‘plant or machine operators’ (8.15 and 7.97/1000 births, respectively), those aged less than 19 or over 40 (7.51 and 7.71/1000 births, respectively), and those who were unmarried (7.63/1000 births) or smoked (6.20/1000 births). Stratification by remoteness status suggested that rates of stillbirth differed minimally by remoteness classification (Table 3).

**Table 3:** Multivariable analysis of risk factors and their association with stillbirth odds in SA, stratified by areas of remoteness (cohort one)

Factors		Adjusted OR for risk factors of stillbirth*	Adjusted OR for risk factors of stillbirth stratified by region of residence*				
			Major city		Inner regional	Outer regional	Remote/very remote area
		aOR (95% CI)	aOR (95% CI)	aOR (95% CI)		aOR (95% CI)	aOR (95% CI)
Smoking	Non-smoker	Referent	Referent	Referent		Referent	Referent
	Smoker	1.13 (0.99, 1.28)	1.16 (0.99, 1.35)	1.28 (0.92, 1.77)		**1.13 (1.00, 1.28)**	0.65 (0.42, 1.00)
Insurance type	Private	Referent	Referent	Referent		Referent	Referent
	Public	1.11 (0.96, 1.28)	1.08 (0.91, 1.28)	1.29 (0.85, 1.96)		1.13 (0.98, 1.31)	1.66 (0.77, 3.57)
Marital status	Married	Referent	Referent	Referent		Referent	Referent
	Unmarried	**1.20 (1.04, 1.39)**	1.18 (0.98, 1.41)	1.41 (0.95, 2.09)		**1.19 (1.02, 1.37)**	1.17 (0.73, 1.90)
Adequate ANC access	Adequate ANC access	Referent	Referent	Referent		Referent	Referent
	Inadequate ANC access	**3.93 (3.41, 4.52)**	**3.53 (2.95, 4.22)**	**5.56 (3.91, 7.92)**		**3.89 (3.38, 4.47)**	**4.64 (2.98, 7.23)**
	12–19 years	1.04 (0.82, 1.32)	0.94 (0.69, 1.28)	0.84 (0.45, 1.59)		1.05 (0.83, 1.33)	1.20 (0.55, 2.61)
Birthing person’s age	20–25 years	0.99 (0.84, 1.16)	1.03 (0.85, 1.26)	0.69 (0.43, 1.11)		0.99 (0.85, 1.17)	1.42 (0.83, 1.43)
	25–29 years	Referent	Referent	Referent		Referent	Referent
	30–34 years	1.01 (0.88, 1.16)	1.00 (0.85, 1.18)	0.96 (0.64, 1.43)		1.00 (0.87, 1.15)	1.11 (0.61, 2.04)
	35–40 years	**1.31 (1.11, 1.54)**	1.15 (0.94, 1.40)	**2.02 (1.34, 3.03)**		**1.29 (1.10, 1.52)**	**1.92 (1.02, 3.64)**
	≥ 40 years	**1.92 (1.50, 2.45)**	**2.02 (1.52, 2.67)**	1.14 (0.48, 2.72)		**1.90 (1.49, 2.43)**	< 5 SBs
Birthing person’s occupation	Professionals	Referent	Referent	Referent		Referent	Referent
	Managers/Admin	1.00 (0.77, 1.31)	1.18 (0.87, 1.60)	0.80 (0.40, 1.61)		1.04 (0.40, 2.69)	< 5 SBs
	Paraprofessionals	1.09 (0.83, 1.43)	1.28 (0.94, 1.73)	0.72 (0.32, 1.64)		< 5 SBs	< 5 SBs
	Tradespersons	1.04 (0.74, 1.48)	1.09 (0.71, 1.66)	1.46 (0.69, 3.10)		< 5 SBs	< 5 SBs
	Clerks	0.94 (0.74, 1.18)	1.03 (0.79, 1.36)	0.64 (0.31, 1.31)		1.06 (0.45, 2.50)	0.67 (0.26, 1.72)
	Sales and service workers	1.04 (0.84, 1.30)	1.11 (0.86, 1.45)	0.78 (0.42, 1.45)		1.56 (0.73, 3.36)	0.49 (0.19, 1.27)
	Plant and machine operators	**1.99 (1.16, 3.43)**	**2.76 (1.55, 4.90)**	< 5 SBs		< 5 SBs	< 5 SBs
	Labourers	1.08 (0.78, 1.49)	1.02 (0.67, 1.54)	0.84 (0.35, 2.04)		2.09 (0.85, 5.10)	< 5 SBs
	Student	1.28 (0.94, 1.75)	1.28 (0.89, 1.94)	1.83 (0.75, 4.45)		< 5 SBs	< 5 SBs
	Pensioner	1.55 (0.80, 3.01)	0.94 (0.34, 2.60)	< 5 SBs		< 5 SBs	< 5 SBs
	Home duties	1.21 (0.98, 1.49)	1.26 (0.98, 1.62)	1.10 (0.64, 1.88)		1.22 (0.57, 2.62)	1.13 (0.55, 2.31)
	Unemployed	**1.34 (1.01, 1.76)**	1.32 (0.93, 1.86)	1.14 (0.52, 2.50)		1.59 (0.63, 4.03)	1.35 (0.54, 3.39)
Non-birthing person occupation	Professionals	Referent	Referent	Referent		0.75 (0.29, 1.92)	< 5 SBs
	Managers/Admin	0.90 (0.72, 1.11)	0.86 (0.66, 1.11)	1.10 (0.60, 2.02)		Referent	Referent
	Paraprofessionals	0.92 (0.68, 1.24)	0.89 (0.63, 1.25)	1.53 (0.69, 3.41)		< 5 SBs	< 5 SBs
	Tradespersons	0.97 (0.78, 1.19)	0.93 (0.73, 1.18)	0.86 (0.46, 1.64)		0.82 (0.46, 1.48)	**1.69 (1.17, 6.16)**
	Clerks	1.26 (0.90, 1.77)	1.30 (0.90, 1.88)	1.78 (0.65, 4.90)		< 5 SBs	< 5 SBs
	Sales and service workers	0.95 (0.72, 1.26)	0.95 (0.69, 1.30)	0.59 (0.21, 1.64)		1.30 (0.57, 2.96)	< 5 SBs
	Plant and machine operators	0.93 (0.71, 1.23)	0.88 (0.63, 1.23)	1.10 (0.52, 2.30)		0.70 (0.30, 1.61)	< 5 SBs
	Labourers	0.96 (0.77, 1.20)	0.91 (0.70, 1.18)	0.81 (0.40, 1.65)		1.11 (0.64, 1.90)	
	Student	1.11 (0.76, 1.63)	1.19 (0.80, 1.77)	< 5 SBs		< 5 SBs	< 5 SBs
	Pensioner	**1.83 (1.12, 2.99)**	**2.01 (1.14, 3.54)**	< 5 SBs		< 5 SBs	< 5 SBs
	Home duties	0.61 (0.23, 1.65)	0.44 (0.11, 1.82)	< 5 SBs		< 5 SBs	< 5 SBs
	Unemployed	**1.33 (1.01, 1.76)**	1.19 (0.85, 1.67)	1.61 (0.73, 3.57)		1.39 (0.71, 2.69)	**4.06 (1.41, 11.73)**
Interpregnancy interval	> 6 months	Referent	Referent	Referent		Referent	Referent
	< 6 months	1.05 (0.85, 1.29)	1.18 (0.92, 1.52)	0.82 (0.46, 1.47)		1.05 (0.85, 1.29)	0.70 (0.32, 1.55)
Country of birth**	Australia	Referent	Referent	Referent		NA	NA
	Europe/USSR	0.90 (0.71, 1.13)	0.92 (0.71, 1.18)	1.10 (0.59, 2.05)		< 5 SBs	< 5 SBs
	Middle east/Nth Africa	1.17 (0.53, 2.63)	1.29 (0.58, 2.89)	< 5 SBs		< 5 SBs	< 5 SBs
	SE Asia	0.84 (0.64, 1.12)	0.89 (0.66, 1.19)	< 5 SBs		< 5 SBs	< 5 SBs
	NE Asia	0.77 (0.50, 1.18)	0.76 (0.47, 1.20)	< 5 SBs		< 5 SBs	< 5 SBs
	Southern Asia	**1.58 (1.19, 2.10)**	**1.64 (1.21, 2.21)**	< 5 SBs		< 5 SBs	< 5 SBs
	Nth America	0.67 (0.04, 2.11)	0.72, 0.27, 1.93)	< 5 SBs		< 5 SBs	< 5 SBs
	South/Central America	0.29 (0.04, 2.11)	0.32 (0.45, 2.30)	< 5 SBs		< 5 SBs	< 5 SBs
	Africa	0.82 (0.29, 2.27))	0.85 (0.26, 2.74)	< 5 SBs		< 5 SBs	< 5 SBs
	Oceania	0.57 (0.30, 1.07)	0.64 (0.31, 1.28)	< 5 SBs		< 5 SBs	< 5 SBs
Ethnicity	Caucasian	Referent	Referent	Referent		Referent	Referent
	Aboriginal/Torres Strait Islander	**1.50 (1.20, 1.88)**	1.26 (0.90, 1.75)	**1.91 (1.06, 3.46)**		**1.55 (1.25, 1.93)**	**1.90 (1.12, 3.21)**
	Asian	1.12 (0.93, 1.35)	1.17 (0.96, 1.42)	< 5 SBs		< 5 SBs	< 5 SBs
Parity	Nulliparous	1.03 (0.90, 1.17)	1.00 (0.85, 1.17)	1.19 (0.85, 1.69)		1.02 (0.89, 1.16)	1.12 (0.69, 1.82)
	Multiparous	Referent	Referent	Referent		Referent	Referent
Remoteness	Major City	Referent	NA	NA		NA	NA
	Inner regional area	1.01 (0.93, 1.27)	NA	NA		NA	NA
	Outer regional area	**1.31 (1.10, 1.55)**	NA	NA		NA	NA
	Remote/Very remote area	1.11 (0.91, 1.37)	NA	NA		NA	NA
Anaemia	No anaemia	Referent	Referent	Referent		Referent	Referent
	Anaemia	0.99 (0.82, 1.18)	0.96 (0.78, 1.20)	1.17 (0.70, 1.94)		0.97 (0.81, 1.17)	1.02 (0.55, 1.87)

aOR adjusted for year, adequate ANC access, marital status, smoking status, parity, remote/rural status, birthing person age, previous stillbirth, maternal ethnicity, medical conditions (pre-existing diabetes or hypertension, anaemia), plurality, interpregnancy interval, insurance status, obstetric complications (gestational diabetes, gestational hypertension, APH).

*aOR adjusted for year, adequate ANC access, marital status, ethnicity, smoking status, parity, maternal age, previous stillbirth, medical conditions (pre-existing diabetes or hypertension, anaemia), plurality, interpregnancy interval, insurance status, obstetric complications (gestational diabetes, gestational hypertension, APH).

** model of adjustment excluding ethnicity.

### Adequacy of antenatal care access (ANC)

Crude analysis demonstrated a fourfold increase in stillbirth odds for birthing people who received inadequate ANC compared with those who received adequate ANC (Table 2). This increased odds of stillbirth following inadequate ANC access was observed across all areas of residence (Table 3). Adjusted analysis demonstrated that birthing people in SA who experienced inadequate versus adequate ANC access had fivefold greater odds of stillbirth (inner region: aOR 5.56; 95% CI 3.91–7.92; remote/very remote region: aOR 4.64; 95% CI 2.98–7.23).

### Parental occupation

Crude analysis indicated that several occupations were associated with stillbirth. Through multivariable analysis, birthing people who worked as plant/machine operators had almost double the odds of stillbirth versus professionals (aOR 1.99; 95% CI 1.16–3.43). Compared with professionals, unemployed birthing people also had increased odds of stillbirth (aOR 1.34; 95% CI 1.01–1.79). No clear differences were noted in the area stratified analysis considering unemployment (compared with major cities, outer regional areas: aOR 1.59; 95% CI 0.63–4.03, remote/very remote areas: aOR 1.35; 95% CI 0.54–3.39).

Unemployed non-birthing parent status (aOR 1.33; 95% CI 1.01–1.76) and pensioner status (aOR 1.83; 95% CI 1.12–2.99) versus professional status were associated with increased odds of stillbirth. Non-birthing parent tradeperson status (aOR 1.69; 95% CI 1.17–6.16) and unemployment (aOR 4.06; 95% CI 1.41–11.73) was independently associated with stillbirth within remote/very remote areas of SA (Table 3).

### Birthing persons’ country of birth

Crude analysis demonstrated increased odds of stillbirth for birthing people born in Southern Asia, the Middle East/North Africa, and Africa versus Australia (Table 2). Increased odds of stillbirth were shown for birthing people from Southern Asia (versus Australia) (aOR 1.58; 95% CI 1.19–2.10). This was mirrored for South Asian-born birthing people residing in major cities (Table 3). Crude analysis revealed greater stillbirth odds for birthing people born in Middle Eastern/North African countries; however, this increase was attenuated in multivariable analyses. Similar results were shown for birthing people from African countries (64% increased odds of stillbirth) (versus Australia); however, the odds were attenuated in the multivariate analysis (aOR 0.82; 95% CI 0.29–2.27). The odds of stillbirth did not increase for any of the other countries in which the birthing people were born compared with those for which the birthing people were born in Australia.

### Birthing persons’ ethnicity

Aboriginal and/or Torres Strait Islander status (versus Caucasian status) was shown to increase stillbirth odds through crude and adjusted analyses (cOR 2.55; 95% CI 2.11–3.08, and aOR 1.50; 95% CI 1.20–1.88). Stratification by place of residence revealed that the odds of stillbirth for Aboriginal and/or Torres Strait Islander versus Caucasian people were almost double within inner regional (aOR 1.91; 95% CI 1.06–3.46) and remote/very remote areas (aOR 1.90; 95% CI 1.12–3.21). Self-reported Asian ethnicity (versus Caucasian status) did not show an increase in stillbirth odds (aOR 1.12; 95% CI 0.93–1.35). Stratification by areas of remoteness could not be performed due to small case numbers per subgroup.

### BMI (cohort two)

Similar to cohort one, analyses of cohort two demonstrated increased odds of stillbirth with inadequate ANC access, particular parental occupations, and certain birthing person’s country of birth and ethnicity (Table 4). Birthing person BMI was associated with marginally increased odds of stillbirth for BMI’s between 35 and 39 at the first antenatal appointment (Table 5). These findings were mirrored through remoteness stratification analysis. The odds of stillbirth were not significant for morbidly obese birthing people according to the analysis, possibly reflecting an underpowered sample size in this category. Through models adjusted for BMI, the associations between Aboriginal and/or Torres Strait Islander ethnicity and stillbirth odds decreased, eliminating this factor’s independent association with stillbirth.

**Table 4:** Multivariable analysis of risk factors and their association with stillbirth odds in SA, between 2007 and 2016 (cohort two)

Variables		Adjusted OR
Smoking	Non-smoker	Referent
	Smoker	1.16 (0.95, 1.42)
Insurance type	Private	Referent
	Public	0.82 (0.65, 1.03)
Marital Status	Married	Referent
	Unmarried	1.23 (0.97, 1.55)
Adequate ANC access	Adequate antenatal care access	Referent
	Inadequate antenatal care access	**4.02 (3.19, 5.06)**
Birthing peron’s age	12–19 years	1.20 (0.81, 1.78)
	20–24 years	0.98 (0.76, 1.27)
	25–29 years	Referent
	30–34 years	1.07 (0.96, 1.32)
	35–39 years	1.17 (0.90, 1.51)
	≥ 40 years	**2.00 (1.40, 2.86)**
Birthing person’s occupation	Professionals/Managers/Admin	Referent
	Clerks/Sales people	1.02 (0.81, 1.29)
	Tradespersons/Labourers/Lab & machine operators	1.08 (0.75, 1.56)
	Student	1.45 (0.97, 2.17)
	Unemployed/Pensioner/Home duties	1.19 (0.93, 1.53)
Non-birthing person’s occupation	Professionals/Managers/Admin	Referent
	Clerks/Salespeople	1.02 (0.73, 1.43)
	Tradespersons/Labourers/Lab & machine operators	1.10 (0.89, 1.36)
	Student	1.13 (0.66, 1.93)
	Unemployed/Pensioner/Home duties	**1.46 (1.04, 2.07)**
Interpregnancy interval	> 6 months	Referent
	≤ 6 months	1.21 (0.90, 1.62)
Birthing person’s country of birth*	Australia	Referent
	Europe/USSR	1.14 (0.80, 1.63)
	Middle east/Nth Africa	**1.87 (1.23, 2.83)**
	SE Asia	0.93 (0.62, 1.40)
	NE Asia	0.90 (0.54, 1.50)
	Southern Asia	**1.67 (1.24, 2.24)**
	Nth America	< 5 SBs
	South/Central America	< 5 SBs
	Africa	**1.96 (1.30, 2.97)**
	Oceania	< 5 SBs
Birthing person’s ethnicity	Caucasian	Referent
	Aboriginal/Torres Strait Islander	1.17 (0.80, 1.72)
	Asian	**1.43 (1.13, 1.82)**
Parity	Nulliparous	0.80 (0.65, 1.00)
	Multiparous	Referent
Remoteness	Major City	Referent
	Inner regional area	1.08 (0.83, 1.40)
	Outer regional area	1.30 (0.97, 1.75)
	Remote/Very remote area	1.36 (0.96, 1.91)
Anaemia	No anaemia during pregnancy	Referent
	Anaemia during pregnancy	1.17 (0.91, 1.52)

aOR adjusted for year of birth, adequate ANC access, marital status, birthing person BMI, maternal ethnicity, smoking status, parity, remote/rural status, birthing person age, previous stillbirth, medical conditions (pre-existing diabetes or hypertension, anaemia), plurality, interpregnancy interval, insurance status, obstetric complications (gestational diabetes, gestational hypertension, APH).

*birthing person ethnicity excluded from model of adjustment.

**Table 5:** Multivariable analysis of risk factors and their association with stillbirth odds in SA, between 2007 and 2016, stratified by areas of remoteness (cohort two)

Birthing person’s BMI category	Total births	Stillbirth rate/1000 births	Crude OR (95% CI)	Adjusted OR for risk factors of stillbirth*	Adjusted OR for risk factors of stillbirth stratified by region of residence**			
					Major city†	Inner regional†	Outer regional†	Remote/very remote†
Underweight (< 19)	5,421	3.49	0.80 (0.50, 1.27)	0.72 (0.44, 1.19)	0.66 (0.08, 5.17)	Referent	Referent	Referent
Healthy weight (19–24)	67,664	4.37	Referent	Referent	Referent			
Overweight (25–29)	45,594	4.32	0.99 (0.82, 1.19)	1.02 (0.84, 1.24)	1.44 (0.82, 2.54)	0.96 (0.76, 1.20)	1.23 (0.60, 2.52)	1.10 (0.54, 2.26)
Obese class 1 (30–34)	22,518	4.38	1.15 (0.96, 1.38)	1.06 (0.82, 1.36)	1.06 (0.50, 2.23)	**1.33** **(1.05, 1.68)**	1.39 (0.67, 2.86)	0.69 (0.28, 1.70)
Obese class 2 (35–39)	10,426	6.01	**1.38 (1.04, 1.83)**	**1.48 (1.08, 2.02)**	1.24 (0.49, 3.12)			
Morbidly Obese (40+)	6,750	5.60	1.28 (0.91, 1.08)	1.29 (0.89, 1.87)	0.99 (0.34, 2.92)			
Missing	42,228	4.83	NA	NA	NA	NA	NA	NA

* aOR adjusted for year of birth, adequate ANC access, marital status, rural/remote status, maternal ethnicity, smoking status, parity, maternal age (< 35, 35–39, > 40), previous stillbirth, medical conditions (pre-existing diabetes or hypertension, anaemia), plurality, interpregnancy interval, insurance status, obstetric complications (gestational diabetes, gestational hypertension, APH).

** aOR adjusted for year, adequate ANC access, marital status, smoking status, parity, maternal age, previous stillbirth, medical conditions (pre-existing diabetes or hypertension, anaemia), plurality, interpregnancy interval, insurance status, obstetric complications (gestational diabetes, gestational hypertension, APH).

†stratified analysis conducted using populations designated as living within a major city (*n* = 110,075 (407 stillbirths)), Inner regional area (*n* = 19,569 (73 stillbirths)), outer regional area (*n* = 11,363 (51 stillbirths), or remote/very remote area (*n* = 7795 (40 stillbirths)). Due to cohort size, BMI categories were grouped (healthy (BMI < 25), overweight (BMI 25–29), obese (BMI > 30)).

### Population attributable fractions (PAFs) (Table 6)

**Table 6:** Multivariable analysis for select risk factors for birthing people residing in South Australia between 1998 and 2016, the population attributable fractions (PAF), and attributable stillbirths* (cohort one)

Variables		aOR (95% CI)	PAF (%)**	Total preventable SB for study period (1998–2016) (births)	Average preventable SB per year in SA (births)
Smoking status	Non-smoker	Referent	.	.	
	Smoker	1.13 (0.99, 1.28)	3.31%	52	3
Adequate ANC access	Adequate ANC access	Referent	.	.	
	Inadequate ANC access	3.93 (3.41, 4.52)	27.65%	437	24
Birthing person’s age	≤ 35 years	Referent	.	.	
	> 35 years	1.40 (1.23, 1.60)	6.32%	100	6
Birthing person’s occupation	All other occupations	Referent	.	.	
	Plant or machine operators	1.74 (1.04, 2.91)	0.40%	6	0.3
Birthing person’s country of birth	All other countries (excluding only population of interest below)	Referent	.	.	
	Southern Asian countries	1.64 (1.23, 2.18)	1.33%	21	1
	African countries	1.55 (1.21, 1.99)	1.52%	24	1
Remoteness	Major city/inner regional	Referent	.	.	
	Outer regional/remote/very remote	1.23 (1.08, 1.41)	3.24%	51	3

*SB = stillbirths, Remoteness = remoteness classification of the maternal residential postcode at the time of birth, aOR = adjusted odds ratio, odds adjusted for year of birth, adequate ANC access, marital status, smoking status, parity, remoteness, maternal age, maternal pre-existing medical conditions (diabetes, hypertension, anaemia), insurance status, interpregnancy interval, plurality, gestational diabetes or hypertension, antepartum haemorrhage (adjustments of individual factors exclude the factor of interest within adjustment).

**PAF calculated using methods described by Mansournia et al. [13].

Factors with the strongest independent associations with stillbirth odds were selected to determine PAFs (Table 6). The PAF enabled examination of the direct percentage of stillbirths attributed to each risk factor within the population according to the populational prevalence. The factors with the greatest impacts on stillbirth rates in SA were inadequate ANC access (PAF: 27.65%) and birthing person age > 35 years (PAF: 6.32%). The PAFs for smoking or residing in outer regional/remote or very remote areas were 3.31% and 3.24%, respectively.

## Discussion

Adequate ANC access in Australia has been highlighted as a marker of inequity between areas of remoteness and major cities [3] and is well established as the best means to ensure a healthy pregnancy and effective preventative care for poor pregnancy outcomes. Our results suggest that inadequate ANC access (as per the Australian pregnancy care guidelines [13]) is strongly associated with increased odds of stillbirth. The recommended number of ANC visits is 10 for first pregnancies and seven for subsequent uncomplicated pregnancies [13]. PAF calculations indicated that if all recommended appointments were accessible to all birthing people, 437 stillbirths could have been prevented over this study period, equating to an average of 24 stillbirths per year. Previous research examining the impact of ANC on stillbirths has revealed a U-shaped curve and has suggested that 14 visits is optimal to minimise risk [14]. Globally, there are notable variations in the minimum number of visits recommended; German studies suggest 12 [15], USA, 11 [16], and Canada [17, 18]. Strategies to encourage improved ANC access, such as culturally safe care models, and addressing travel and financial barriers to access, alongside further consideration of an increase in the minimum number of recommended ANC visits in Australia, should constitute part of stillbirth prevention efforts.

Remote and rural status has previously been shown to have an independent association with intrapartum stillbirth in remote Western Australia due to a lack of access to high-level care during labour, although Aboriginal and/or Torres Strait Islander women were excluded from these findings because the main outcome focused on migrant women in Western Australia [19]. Comparable results have been shown in studies examining the impact of regional and remote living on stillbirth rates in Australia [20, 21], although the findings were limited by cohort size and limited confounder adjustment. Our analysis revealed marginally greater odds of stillbirth within regional areas (i.e., the outer and inner regional areas), and for birthing people who smoked during pregnancy, who were unmarried or of advanced age (over 35 years). Aboriginal and/or Torres Strait Islander birthing people were at increased risk of stillbirth in inner and outer regional areas. These findings further highlight the need for increased preventative care for those living in regional and remote areas.

There are mixed findings regarding the association between Aboriginal and/or Torres Strait Islander People and stillbirth odds. Some have reported increased odds of stillbirth, while others have reported equivalence [22, 23]. Our study suggested that Aboriginal and/or Torres Strait Islander birthing people are at risk of 21 stillbirths per year in SA. Compared with Caucasian birthing people, Aboriginal and/or Torres Strait Islander birthing people residing in inner regional and remote/very remote areas experience greater stillbirth odds than their city-dwelling counterparts. An analysis incorporating BMI into models of adjustment diminished this association, indicating that there was no independent association with stillbirth odds and that strategies to address BMI may be key. This may implicate a combined lack of culturally safe care models, limited birthing on country services, and poorly resourced ANC in regional and remote areas of SA. Cultural safety and birthing on country training of health care professionals has been shown to improve access for Aboriginal and/or Torres Strait Islander families, including trauma-informed care [24].

South Asian ethnicity has previously been shown to have an independent association with stillbirth odds in HIC populations globally [19, 25–30]. Analyses of stillbirth odds for birthing people of South Asian ethnicity have differed when country of birth has been used as a proxy for ethnicity in previous studies [19, 22, 26–28, 31] versus when self-reported ethnicity has been used [25, 29, 30]. Although country of birth is a commonly used proxy for ethnicity in some studies, there is a need for clear differentiation, as these are two different variables. One captures migration status, and the other captures self-reported ethnicity. The findings of this study demonstrate that South Asian (versus Australian) countries of birth are associated with stronger odds of stillbirth than self-reported Asian (versus Caucasian) ethnicity. Country of birth should be considered an independent factor when assessing the risk of stillbirth at the individual level.

Certain occupations and their associated exposures to chemicals or lifting and rotating shift work have previously been implicated as contributors to stillbirth in HICs [32–34]. To our knowledge, there has been no prior research examining associations between stillbirth and occupational groups within an entire population. The increased odds of stillbirth for plant- or machine-operating birthing people warrants attention. As does paternal unemployment and tradesperson status in remote and very remote areas. – both also associated with increased stillbirth odds in SA.

According to previous research on HICs, obesity consistently and independently increases stillbirth odds [31, 35, 36]. Our findings demonstrated that a BMI between 35 and 39 was associated with increased odds of stillbirth, but this was not observed when the BMI reached ≥ 40. This observation may be due to the low number of birthing people with a BMI ≥ 40, rendering the analysis underpowered. However, the absence of increased stillbirth odds for birthing people with a BMI ≥ 40 could be due to the different care pathways and tailored care and monitoring for this group. In SA, at their first antenatal appointment, this group is provided specific ANC programs focused on pregnancy risks and complications associated with morbid obesity [37].

### Strengths and limitations

The strengths of this study lie in the comprehensive and detailed measures for each birth, including the inclusion of parental occupational coding and ethnicity alongside country of birth. Factors included within this dataset are collected routinely for the entire study period without changes in the definition or classification of diseases. Due to the large number of stillbirths included in this study, analysis of many factors was possible, allowing meaningful and generalisable results. However, we acknowledge several limitations. The omission of BMI data collected prior to 2007 prevented the analysis of BMI across the study period. Cohort two encompasses BMI, but due to the smaller cohort size, comprehensive analysis was not possible. This study has the same limitations ubiquitous to research examining routinely collected perinatal data, which may not have been intended solely for research purposes. The lack of data concerning domestic assault, pollution, consanguinity, sleep position and drug/alcohol use leaves potential for residual bias due to unmeasured covariates. The current analysis does not account for the temporal changes in individual factors’ impacts over the course of the study period. The use of average ARIA + scores from SA2’s encompassed within each SA3 for remoteness status has the potential to result in misclassification of remoteness status for some populations within assigned categories.

## Conclusion

Results demonstrate gaps in national- and state/territory-level analysis of stillbirth in Australia. Our findings indicate that inadequate ANC access is the greatest risk factor for stillbirth in SA, particularly remote SA. Complexities preventing engagement in care and poor attendance may reflect access to and acceptability of ANC programs across all facets of society. The evidence presented indicates that further research is needed to determine the required minimum number of ANC visits and provision of adequate access to the recommended number of ANC visits for all birthing people. This also needs to take into consideration the implications for current health care systems, especially in remote and regional areas. Omission of stratification by residential remoteness in previous research has masked disparities between marginalised groups within regions that are shown to have the highest rates of stillbirth. Through stratification, this research identifies that different factors are associated with increased stillbirth odds for people living in regional and remote areas of South Australia, than those living in major cities. The lack of evidence for these differences previously has meant that current pregnancy care guidelines and policies, although based on evidence at the national level, do not address differences that exist for health care providers serving populations in regional and remote areas, which are overlaid with finite access to resources. It is clear from our findings that the stillbirth odds for birthing people aged 35–40 years or with specific occupations differ according to residential remoteness classification. Through robust sub analysis incorporating comprehensive multivariable adjustment (including BMI), our findings demonstrate that birthing person Aboriginal and/or Torres Strait Islander status is not independently associated with stillbirth, and while there is no independent association, holistic and culturally safe care is essential. Improved access to care will aid in addressing factors that may be independently associated with and contributing to stillbirth rates within this population.

### Electronic supplementary material

Below is the link to the electronic supplementary material.

Supplementary Material 1

## Data Availability

The deidentified data analysed are not publicly available, but requests to the corresponding author for the data will be considered on a case-by-case basis in discussion with the South Australian Data Custodian. Requests may be referred to the South Australian Data Custodian to obtain approval.
