# Supplementary material for: Lifestyle and sociodemographic risk factors for stillbirth by region of residence in South Australia: a retrospective cohort study
Source: BMC Pregnancy Childbirth. 2024 May 15;24:368. doi: 10.1186/s12884-024-06553-5 (PMC11097586; doi:10.1186/s12884-024-06553-5)
Supplement: Supplementary file 1 — Supplementary Material 1 [file 12884_2024_6553_MOESM1_ESM.docx]

Supplementary table: STROBE Statement— Lifestyle and sociodemographic risk factors for stillbirth by region of residence in South Australia: a retrospective cohort study

|  | | | Item No. | Recommendation | | Page  No. | | Relevant text from manuscript | | |  |
| --- | --- | --- | --- | --- | --- | --- | --- | --- | --- | --- | --- |
| **Title and abstract** | | | 1 | (*a*) Indicate the study’s design with a commonly used term in the title or the abstract | | 1 | | *Retrospective cohort study.* | | |  |
|  |  |  |  | (*b*) Provide in the abstract an informative and balanced summary of what was done and what was found | | 1 | | *Associations between stillbirth and lifestyle and sociodemographic factors were evaluated using multivariable logistic regression and described using adjusted odds ratios (aOR).* | | |  |
| Introduction | | | | | | | |  | | |  |
| Background/rationale | | | 2 | Explain the scientific background and rationale for the investigation being reported | | 3 | | *Rumbold et al (5) highlighted the impact of inequity on stillbirth rates within select Australian populations, noting particular concern within communities experiencing isolation and socioeconomic disadvantage (5). Numerous risk factors in disadvantaged communities contribute to the widening gap of health inequality, further hindering stillbirth prevention (10).* | | |  |
| Objectives | | | 3 | State specific objectives, including any prespecified hypotheses | | 3 | | *This research aims to identify lifestyle and sociodemographic risk factors for stillbirth in South Australia (SA), geographical and to explore these risks according to remoteness* | | |  |
| Methods | | | | | | | |  | | |  |
| Study design | | | 4 | Present key elements of study design early in the paper | | 3 | | *This is a retrospective state-wide observational cohort study using the SA Perinatal outcomes dataset, including all births from 1998 to 2016 inclusive* | | |  |
| Setting | | | 5 | Describe the setting, locations, and relevant dates, including periods of recruitment, exposure, follow-up, and data collection | | 3 | | *State-wide observational cohort study using the SA Perinatal outcomes dataset, including all births from 1998 to 2016 inclusive… In SA, all births are reported by midwives, birth attendants and obstetricians on standardised supplementary birth records. The SA Perinatal Outcomes Unit integrate continuous validation of the dataset by comparing data collected on the supplementary birth record to electronic hospital records at the time of coding.* | | |  |
| Participants | | | 6 | (*a*) *Cohort study*—Give the eligibility criteria, and the sources and methods of selection of participants. Describe methods of follow-up  *Case-control study*—Give the eligibility criteria, and the sources and methods of case ascertainment and control selection. Give the rationale for the choice of cases and controls  *Cross-sectional study*—Give the eligibility criteria, and the sources and methods of selection of participants | | 3 | | *This is a retrospective state-wide observational cohort study using the SA Perinatal outcomes dataset, including all births from 1998 to 2016 inclusive*  *Terminations of pregnancy were excluded.* | | |  |
|  |  |  |  | (*b*) *Cohort study*—For matched studies, give matching criteria and number of exposed and unexposed  *Case-control study*—For matched studies, give matching criteria and the number of controls per case | | Table 2 | | *Number of unexposed vs exposed are included in table 2* | | |  |
| Variables | | | 7 | Clearly define all outcomes, exposures, predictors, potential confounders, and effect modifiers. Give diagnostic criteria, if applicable | | 3 | | *Variable definitions and time periods are provided in table 1. Information for all births (live or stillborn) ≥ 20 weeks’ gestational age (GA), or ≥ 400 grams at birth are reported. The primary outcome, stillbirth, was defined in line with the standard Australian Institute of Health and Wellbeing definition as the birth of a baby showing no signs of life at ≥ 20 weeks’ completed GA, or ≥ 400 grams birthweight where no GA is provided.* | | |  |
| Data sources/ measurement | | | 8* | For each variable of interest, give sources of data and details of methods of assessment (measurement). Describe comparability of assessment methods if there is more than one group | | Table 1 | | *See table 1 for details on each variable, exposure and outcome* | | |  |
| Bias | | | 9 | Describe any efforts to address potential sources of bias | | 9 | | *Despite the strengths that lay in sue foal l available confounders within the dataset, Lack of data concerning domestic assault, pollution, consanguinity, sleep position and drug/alcohol use leaves potential for residual bias due to unmeasured covariates.* | | |  |
| Study size | | | 10 | Explain how the study size was arrived at | | 3 | | *All available births were used from the beginning of SA perinatal dataset collection. Where sample size was inadequate for analysis, or small numbers rendered the data identifiable, analysis was not performed as per the instructions from the author biostatistician.* | | |  |
| Quantitative variables | | 11 | | | Explain how quantitative variables were handled in the analyses. If applicable, describe which groupings were chosen and why | | Table 1 | | | See table 1 | |
| Statistical methods | | 12 | | | (*a*) Describe all statistical methods, including those used to control for confounding | | 4 | | | *Unadjusted and adjusted models were considered, with adjustment made for variables that demonstrated significance during univariate analysis (p < 0.001). For each risk factor, adjustment variables included year of birth, adequate antenatal care (ANC) access (adjusted for GA at birth), marital status, ethnicity, smoking status, parity, remote/rural status, age, previous stillbirth, medical conditions (pre-existing diabetes, hypertension, anaemia), plurality, interpregnancy interval, insurance status, obstetric complications (gestational diabetes, gestational hypertension, antepartum haemorrhage (APH)). The cohort was stratified by residential remoteness and analysis repeated using the same adjustment variables (excluding rural/remote status). Factors demonstrating the strongest association with stillbirth odds were further explored to calculate SA specific population attributable fractions (12) and annual attributable stillbirths per factor (n). Analysis was repeated for cohort two, additionally adjusted for BMI (tables 4 and 5).* | |
|  |  |  |  |  | (*b*) Describe any methods used to examine subgroups and interactions | | 4 | | |  |  |
|  |  |  |  |  | (*c*) Explain how missing data were addressed | | 4 | | | *Where exposure data, or variable data was missing, individual births were excluded from analysis.* | |
|  |  |  |  |  | (*d*) *Cohort study*—If applicable, explain how loss to follow-up was addressed  *Case-control study*—If applicable, explain how matching of cases and controls was addressed  *Cross-sectional study*—If applicable, describe analytical methods taking account of sampling strategy | |  | | | NA | |
|  |  |  |  |  | (*e*) Describe any sensitivity analyses | |  | | | NA | |
| Results | | | | | | | | | | | |
| Participants | | 13* | | | (a) Report numbers of individuals at each stage of study—eg numbers potentially eligible, examined for eligibility, confirmed eligible, included in the study, completing follow-up, and analysed | | Table 2 | | | See table 2 | |
|  |  |  |  |  | (b) Give reasons for non-participation at each stage | |  | | | NA | |
|  |  |  |  |  | (c) Consider use of a flow diagram | |  | | | NA | |
| Descriptive data | | 14* | | | (a) Give characteristics of study participants (eg demographic, clinical, social) and information on exposures and potential confounders | | Page 5, table 2 and 3 | | | *Birthing people were predominantly Australian born (81%) with 86% of Australian born people identifying as Caucasian. The majority (71%) lived in major cities, followed by in inner regional areas (14%), outer regional areas (8%) and remote or very remote areas (6%). During pregnancy, 13.5% of birthing people accessed less than the recommended number of ANC visits (Australian Clinical Practice Guidelines: Pregnancy Care recommends nulliparous women have a minimum of 10, and multiparous women, a minimum of 7). Most birthing people were non-smoking (78%) and gave birth in the Australian public health care system (70%) (table 2). Cohort two included 201,315 births (918 stillbirths) between 2007 and 2016.*  *The stillbirth rate in SA over the study period was 4.85/1000 births. Stillbirth rates were highest for birthing people who had inadequate ANC access (13.78/1000 births), and those who reported that they (8.78/1000 births) or their partner were a ‘pensioner’ (10.21/1000 births). Stillbirths were high among ‘unemployed’ individuals and ‘plant or machine operators’ (8.15 and 7.97/1000 births respectively), those aged less than 19 or over 40 (7.51 and 7.71/1000 births respectively), who were unmarried (7.63/1000 births) or smoked (6.20/1000 births). Stratification by remoteness status suggested that rates of stillbirth differed minimally by remoteness classification (table 3).* | |
|  |  |  |  |  | (b) Indicate number of participants with missing data for each variable of interest | | Table 2 | | | See table 2 | |
|  |  |  |  |  | (c) *Cohort study*—Summarise follow-up time (eg, average and total amount) | | NA | | |  | |
| Outcome data | | 15* | | | *Cohort study*—Report numbers of outcome events or summary measures over time | | *Table 2* | | |  | |
|  |  |  |  |  | *Case-control study—*Report numbers in each exposure category, or summary measures of exposure | | *-* | | |  | |
|  |  |  |  |  | *Cross-sectional study—*Report numbers of outcome events or summary measures | | *-* | | |  | |
| Main results | | 16 | | | (*a*) Give unadjusted estimates and, if applicable, confounder-adjusted estimates and their precision (eg, 95% confidence interval). Make clear which confounders were adjusted for and why they were included | | Table 2 | | |  | |
|  |  |  |  |  | (*b*) Report category boundaries when continuous variables were categorized | | Table 1 | | |  | |
|  |  |  |  |  | (*c*) If relevant, consider translating estimates of relative risk into absolute risk for a meaningful time period | | Table 4, 5, 6 and 7 | | |  | |
| Other analyses | 17 | | Report other analyses done—eg analyses of subgroups and interactions, and sensitivity analyses | | | | Table 3, table 5 | | *Analysis stratification by areas of remoteness* | | |
| *Discussion* | | | | | | | | | | | |
| Key results | 18 | | Summarise key results with reference to study objectives | | | |  | | *Our results suggest that inadequacy of ANC access (as per the Australian pregnancy care guidelines (13)) is strongly associated with increased odds of stillbirth (pg7)*  *Our analysis found marginally higher odds of stillbirth within regional areas (outer and inner regional areas), and for birthing people who smoked during pregnancy, were unmarried, or of advanced age (over 35 years). (pg8)*  *Findings of this study demonstrate that South Asian (versus Australian) country of birth is associated with stronger odds of stillbirth, than self-reported Asian (versus Caucasian) ethnicity. (pg 9)* | | |
| Limitations | 19 | | Discuss limitations of the study, taking into account sources of potential bias or imprecision. Discuss both direction and magnitude of any potential bias | | | | Pg9-10 | | *Strengths and limitations paragraph* | | |
| Interpretation | 20 | | Give a cautious overall interpretation of results considering objectives, limitations, multiplicity of analyses, results from similar studies, and other relevant evidence | | | | Pg 10-11 | | *Conclusion paragraph* | | |
| Generalisability | 21 | | Discuss the generalisability (external validity) of the study results | | | | Pg 10-11 | | Conclusion paragraph | | |
| Other information | | |  | | | | | | | | |
| Funding | 22 | | Give the source of funding and the role of the funders for the present study and, if applicable, for the original study on which the present article is based | | | | Pg 11 | | Funding paragraph | | |

*Give information separately for cases and controls in case-control studies and, if applicable, for exposed and unexposed groups in cohort and cross-sectional studies.

**Note:** An Explanation and Elaboration article discusses each checklist item and gives methodological background and published examples of transparent reporting. The STROBE checklist is best used in conjunction with this article (freely available on the Web sites of PLoS Medicine at http://www.plosmedicine.org/, Annals of Internal Medicine at http://www.annals.org/, and Epidemiology at http://www.epidem.com/). Information on the STROBE Initiative is available at www.strobe-statement.org.
